# Zoonotic intestinal parasites of carnivores: A systematic review in Iran

**DOI:** 10.14202/vetworld.2018.58-65

**Published:** 2018-01-23

**Authors:** Shahabeddin Sarvi, Ahmad Daryani, Mehdi Sharif, Mohammad Taghi Rahimi, Mohammad Hasan Kohansal, Siavash Mirshafiee, Abolghasem Siyadatpanah, Seyed-Abdollah Hosseini, Shirzad Gholami

**Affiliations:** 1 Department of Parasitology and Mycology, Toxoplasmosis Research Center, Mazandaran University of Medical Sciences, Sari, Iran; 2 Department of Parasitology, School of Medicine, Shahroud University of Medical Sciences, Shahroud, Iran; 3 Department of Parasitology and Mycology, School of Medicine, Zanjan University of Medical Sciences, Zanjan, Iran; 4 Department of Husbandry, Qaemshahr branch of Islamic Azad University, Mazandaran, Iran; 5 Department of Parasitology and Mycology, School of Medicine, Mazandaran University of Medical Sciences, Sari, Iran

**Keywords:** carnivores, intestinal parasites, zoonotic disease

## Abstract

**Aim:**

Parasitic infections, especially of the zoonotic-parasitic type, are the most important health, economic, and social problems in developing countries, including Iran. The aim of this study was to review systematically the available data on gastrointestinal parasites of carnivores in Iran and their ability to infect humans.

**Materials and Methods:**

Studies reporting intestinal parasites of carnivores were systematically collected from nine electronic English and Persian databases and Proceedings of Iranian parasitology and veterinary congresses published between 1997 and 2015. A total of 26 studies issued from 1997 to 2015 met the eligibility criteria.

**Results:**

The pooled proportion of intestinal parasites of carnivores was estimated as 80.4% (95% confidence interval=70.2-88.8%). The overall prevalence of gastrointestinal parasites in dogs, cats, foxes, and jackals were 57.89%, 90.62%, 89.17%, and 97.32%, respectively. Dipylidium caninum (20.45%), Toxocara spp. (18.81%), Taenia hydatigena (15.28%), Mesocestoides lineatus (11.83%), Echinococcus granulosus (10%), and Toxascaris leonina (8.69%) were the most frequently observed parasites.

**Conclusion:**

High prevalence rates of zoonotic intestinal parasites of carnivores particularly Echinococcus spp. and Toxocara spp. increase the risk of acquiring zoonotic infections such as cystic hydatid, alveolar cysts, and visceral or ocular larva migrants in Iranian people. Therefore, it is essential for public health centers to develop more effective control strategies to decrease infections rates in carnivores’ populations.

## Introduction

Parasitic infections, particularly those capable of zoonosis, are the most important health, economic, and social problems in developing countries, including Iran. Carnivores are definitive or reservoirs hosts for more than 60 zoonotic parasites [1,2]. The presence of these animals in close contact with people constitutes a high potential risk of infection, especially for children due to their poor hygiene relative to adults and higher exposure to contact with contaminated soil containing parasite eggs or cysts. Further, farmers and ranchers who often work in agriculture and animal husbandry are at risk [1,3]. Several gastrointestinal parasites of canines particularly *Toxocara* spp., *Ancylostoma* spp., *Echinococcus* spp., *Dipylidium* spp., *Giardia*, and *Cryptosporidium* spp. are considered important in the public health area by the zoonotic potential that they present [4,5].

Zoonotic parasites that are transmitted to humans can be divided into four groups: (i) Direct zoonotic parasites that infect humans directly from animals including: *Giardia*, *Cryptosporidium*, and *Toxocara*, (ii) saprozoonotic parasites that are transmitted through soil or water, for example, *Ancylostoma* spp. and *Strongyloides stercoralis*, (iii) meta-zoonotic parasites that humans acquire through invertebrate intermediate hosts including *Fasciola* spp. and *Dipylidium caninum*, and (iv) cyclo-zoonotic parasites that can infect humans through vertebrate intermediate hosts such as *Echinococcus granulosus* and *Taenia* spp. [5-8].

Infections with some of the aforementioned parasites cause symptoms and clinical manifestations in humans including hydatidosis, visceral larva migrans, coenurosis, creeping eruption, mesocestoidiasis, and dipylidiasis [1,9]. Among the above-mentioned zoonotic-parasitic infections, Iran is considered an important endemic area of hydatidosis. In addition, toxocariasis is a zoonotic disease with a documented high prevalence in this country [10]. Although numerous studies have been conducted in relation to the prevalence of gastrointestinal parasites in carnivores in Iran, there is no systematic review to analyze this data. Therefore, the major objective of the current systematic review was to determine the prevalence of gastrointestinal parasites in carnivores, and further, to describe the epidemiological status of zoonotic parasitic infections of carnivores in Iran.

## Materials and Methods

### Database search

**Figure-1:**
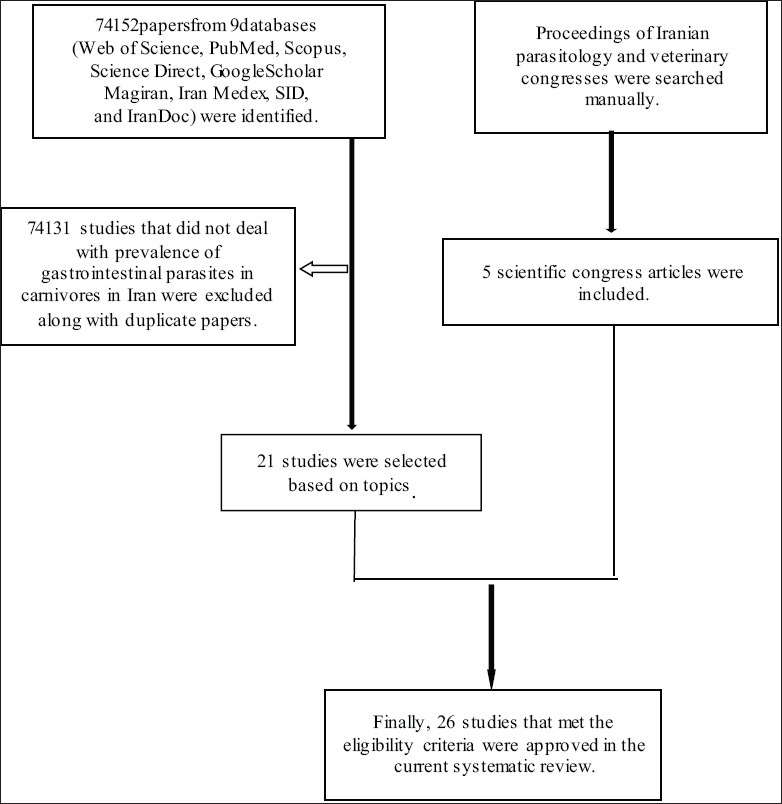
Flow diagram describing the study design process.

Five English databases (PubMed, Scopus, Science Direct, Web of Science (ISI), and Google Scholar) and four Persian databases (Magiran, Scientific Information Database, Iran Medex, and Iran Doc) were searched for published articles about intestinal parasites of carnivores in Iran from 1997 to 2015. The language of data collection was limited to English and Persian. The keywords used alone or in combination were: “Intestinal parasite,” “zoonotic parasites,” “carnivores,” “dogs,” “cats,” “foxes,” “jackals,” “prevalence,” “helminth,” “protozoa,” and “Iran.” All Iranian parasitology and veterinary congresses proceedings were carefully evaluated. To avoid missing any papers, all references from each article were systematically checked (Figure-1).

### Data extractions

Extracted data from the studies included information about the year of publication, first author, province of the study, diagnostic method, sample size, number of positive samples, types of carnivorous, types of gastrointestinal parasites, and types of animal (domestic or stray). In addition, studies which were attributed to human, tissue, blood parasite, case report, and repetitive papers have been excluded.

### Statistical analysis

The pooled proportion of carnivore intestinal parasitic infection, as well as, 95% confidence interval (CI) was calculated for each study. A forest plot was used to visualize heterogeneity among the included studies. The size of every square indicated the weight of every study also the crossed lines illustrated CI. The heterogeneity was expected in advance, and statistical analyses including I^2^ and Cochrane’s Q test (with a significance level of p<0.1) were used to quantify variations. The StatsDirect statistical software (http://www.statsdirect.com) was used for analysis.

## Results

**Figure-2:**
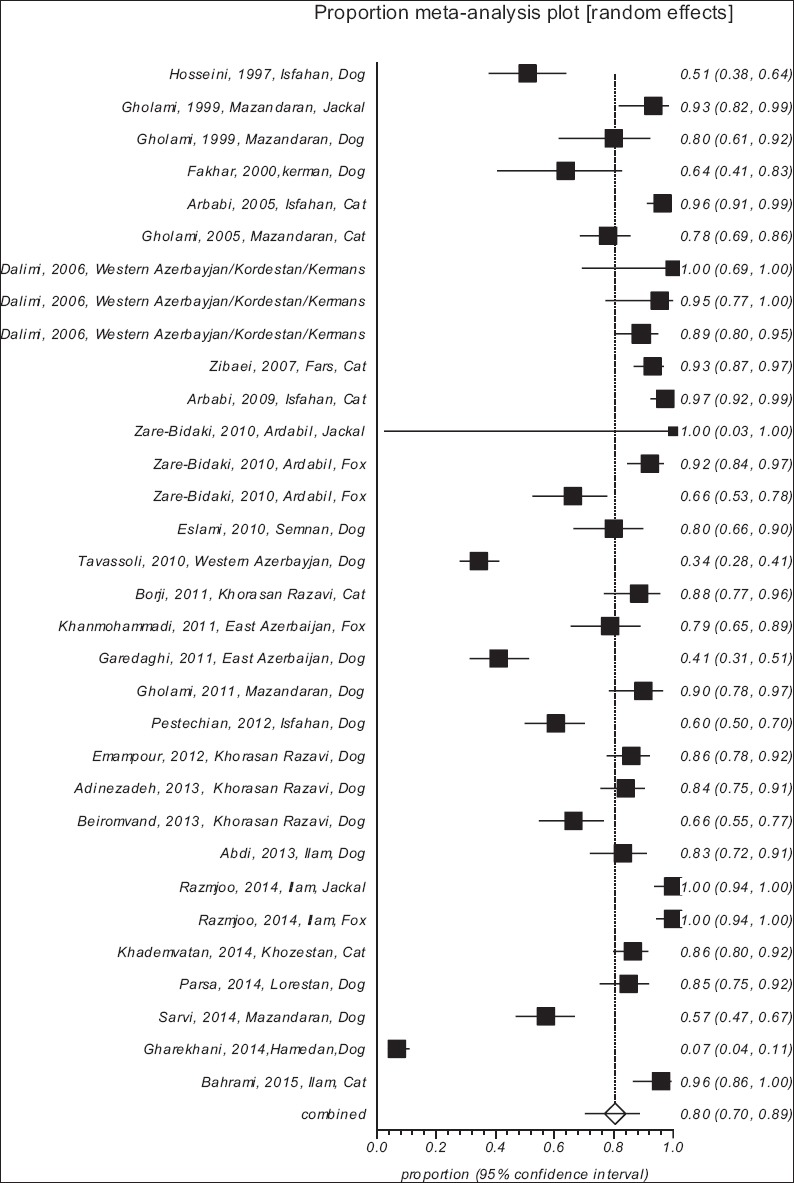
Forest plot diagram of intestinal parasitic infections in carnivores in Iran.

A total of 26 studies in Iran, reporting for carnivorous intestinal parasites, fulfilled our inclusion criteria (Table-1) [11-34]. A total of 2,508 samples were analyzed, and the pooled proportion of carnivore intestinal parasites, in Iran, from 1997 to 2015 was estimated at 80.4% using a random effect model (95% CI=70.2-88.8%) and a forest plot diagram of the study was generated (Figure-2). A wide variation of these parasites was observed in the included studies (Q statistic=1107, degree of freedom (df)=31, p<0.0001, and I²=97.2%). The overall prevalence of gastrointestinal parasite in dogs, cats, foxes, and jackals was 57.89%, 90.62%, 89.17%, and 97.32%, respectively (Table-2).

**Table 1:** Details of the included studies of intestinal parasitic infections in carnivores in Iran.

ID	Province of Iran	Animal	Domestic/Stray	Diagnostic method	Sample size	Parasite positive samples	Prevalence (%)	Ref
1	Isfahan	Dog	Stray	Parasitologic	61	31	50.81	[11]
2	Mazandaran	Jackals	Stray	Parasitologic	45	42	93.33	[12]
		Dog	Stray	Parasitologic	30	24	80	
3	Kerman	Dog	Stray	Parasitologic	22	14	63.63	[13]
4	Isfahan	Cat	Stray	Parasitologic	113	109	96.46	[14]
5	Mazandaran	Cat	Stray	Parasitologic	100	78	78	[15]
6	Western Azerbaijan/Kurdistan/Kermanshah	Jackals	Stray	Parasitologic	10	10	100	[3]
		Fox	Stray	Parasitologic	22	21	95.45	
		Dog	Stray	Parasitologic	83	74	89.15	
7	Fars	Cat	Stray	Parasitologic	114	106	92.98	[16]
8	Isfahan	Cat	Stray	Parasitologic	113	110	97.34	[10]
9	Ardabil	Jackals	Stray	Parasitologic	1	1	100	[17]
		Fox	Stray	Parasitologic	89	82	92.13	
		Dog	Domestic	Parasitologic	59	39	66.1	
10	Semnan	Dog	Stray	Parasitologic	50	40	80	[18]
11	Western Azerbaijan	Dog	Stray	Parasitologic	206	71	34.46	[19]
12	Razavi Khorasan	Cat	Stray	Parasitologic	52	46	88.46	[20]
13	Eastern Azerbaijan	Fox	Stray	Parasitologic	52	41	78.84	[21]
14	Eastern Azerbaijan	Dog	ND	Parasitologic	100	41	41	[22]
15	Mazandaran	Dog	Stray	Parasitologic	50	45	90	[23]
16	Isfahan	Dog	Stray	Parasitologic	96	58	60.4	[24]
17	Razavi Khorasan	Dog	Stray	Parasitologic	100	86	86	[25]
18	Ilam	Dog	Stray	Parasitologic	65	54	83.07	[26]
19	Razavi Khorasan	Dog	Stray	Parasitologic	100	84	84	[27]
20	Razavi Khorasan	Dog	Stray/Domestic	Parasitologic and PCR	77	51	66.23	[28]
21	Ilam	Jackals	Stray	Microscopic	56	56	100	[29]
		Fox	Stray	Parasitologic	62	62	100	
22	Khuzestan	Cat	Stray	Parasitologic	140	121	86.42	[30]
23	Lorestan	Dog	Stray	Parasitologic	80	68	85	[31]
24	Mazandaran	Dog	Stray	Parasitologic and PCR	100	57	57	[32]
25	Hamedan	Dog	Stray	Parasitologic	210	14	6.66	[33]
26	Ilam	Cat	Stray	Parasitologic	50	48	96	[34]

ID=Identification number, Ref=Reference, PCR=Polymerase chain reaction

**Table 2:** Prevalence of gastrointestinal parasitic infections in different species of carnivores in Iran.

Carnivores	Status of gastrointestinal parasites		

	N (%)		

	Uninfected	Infected	Total
Dog	648 (42.11)	891 (57.89)	1539 (100)
Cat	64 (9.38)	618 (90.62)	682 (100)
Fox	30 (10.83)	247 (89.17)	277 (100)
Jackal	3 (2.68)	109 (97.32)	112 (100)

N=Number of samples

The most frequently observed parasite in carnivores was *D. caninum* (20.45%), followed by *Toxocara* spp. (18.81%), *Taenia hydatigena* (15.28%), *Mesocestoides lineatus* (11.83%), *Diplopylidium nolleri* (10.7%), *E. granulosus* (10%), *Joyeuxiella* (9.65%), *Ancylostoma caninum* (8.77%), *Toxascaris leonina* (8.69%), *Rictolaria efinis* (6.66%), *Physaloptera* spp. (6.5%), *Macracanthorhynchus hirudinaceus* (3.1%), *Taenia taeniaeformis* (2.75%), *Uncinaria stenocephala* (2.6%), *Sarcocystis* spp. (1.8%), *Eimeria* spp. (1.4%), and *Trichuris vulpis* (0.57%).

By host species, *T. hydatigena* was the most common parasite (23.91%) in dogs, *Joyeuxiella* (35.04%) in cats, and *M. lineatus* (51.98%) in foxes and (39.28%) jackals (Table-3).

**Table 3:** Prevalence of gastrointestinal parasitic infections among foxes, dogs, cats, and jackals in Iran.

Parasite species	N (%)				

	Dogs (n=1539)	Cats (n=682)	Foxes (n=277)	Jackals (n=112)	Total (n=2610)
*Dipylidium caninum*	260 (16.89)	234 (34.31)	14 (5.05)	26 (23.21)	534 (20.45)
*Mesocestoides lineatus*	99 (6.4)	22 (3.81)	144 (51.98)	44 (39.28)	309 (11.83)
*Toxascaris leonina*	160 (10.39)	10 (1.46)	54 (19.49)	3 (2.67)	227 (8.69)
*Toxocara* spp.	190 (12.34)	179 (26.24)	97 (35.01)	25 (22.32)	491 (18.81)
*Ancylostoma caninum*	97 (6.3)	69 (6.14)	31 (11.19)	32 (28.57)	229 (8.77)
*Taenia hydatigena*	368 (23.91)	2 (0.29)	24 (8.66)	5 (4.46)	399 (15.28)
*Echinococcus granulosus*	225 (14.61)	-	5 (1.8)	12 (10.71)	242 (16)
*Taenia ovis*	54 (3.5)	-	ND (ND)	ND (ND)	54 (2.8)
*Taenia taeniaeformis*	6 (0.38)	47 (6.89)	-	19 (16.96)	72 (2.75)
*Taenia multiceps*	74 (4.8)	-	ND (ND)	1 (0.89)	75 (3.89)
*Uncinaria stenocephala*	18 (1.16)	ND (ND)	17 (6.13)	33 (29.46)	68 (2.6)
*Joyeuxiella*	4 (0.25)	239 (35.04)	6 (2.16)	3 (2.67)	252 (9.65)
*Trichuris vulpis*	6 (0.38)	-	5 (1.8)	ND (ND)	11 (0.57)
*Richtolaria efinis*	21 (1.36)	-	83 (29.96)	70 (62.5)	174 (6.66)
*Physaloptera*	2 (0.13)	147 (21.55)	-	21 (7.58)	170 (6.5)
*Diplopylidium nolleri*	-	73 (10.7)	-	-	73 (10.7)
*Isospora* spp.	5 (0.32)	75 (10.99)	ND (ND)	4 (3.57)	84 (3.21)
*Sarcocystis*	13 (0.84)	35 (5.13)	ND (ND)	ND (ND)	48 (1.83)
*Eimeria*	15 (0.97)	21 (3.07)	3 ND	ND (ND)	39 (1.49)
*Macracanthorhynchus hirudinaceus*	6 (0.39)	ND (ND)	69 (24.9)	6 (5.35)	81 (3.1)

ND=Not detected

According to the study areas, West Azerbaijan/Kurdistan/Kermanshah (89.15%), Isfahan (97.34%) and Ilam (100%) provinces had the highest rate of intestinal parasitic infections in dogs, cats, and foxes (Table-4) [3,13-34]. Moreover, the prevalence of these parasites in jackals in most provinces was 100%.

**Table 4:** Prevalence of gastrointestinal parasitic infections by areas among dogs, cats, foxes, and jackals in Iran.

Province	Dog		Cat		Foxe		Jackal		References
			
	No.	Pos. (%)	No.	Pos. (%)	No.	Pos. (%)	No.	Pos. (%)	
Ardabil	59	39 (66.1)	-	-	89	82 (92.13)	1	1 (100)	[17]
Eastern Azerbaijan	100	41 (41)	-	-	52	41 (78.84)	-	-	[21,22]
Fars	-	-	114	106 (92.98)	-	-	-	-	[16]
Hamedan	210	14 (6.66)	-	-	-	-	-	-	[33]
Ilam	65	54 (83.07)	50	48 (96)	62	62 (100)	56	56 (100)	[26,29,34]
Isfahan	157	89 (56.68)	226	219 (96.9)	-	-	-	-	[14,24]
Kerman	22	14 (63.63)	-	-	-	-	-	-	[13]
Razavi Khorasan	277	221 (79.78)	54	46 (88.46)	-	-	-	-	[20,25,27,28]
Khuzestan	-	-	140	121 (86.42)	-	-	-	-	[30]
Lorestan	80	68 (85)	-	-	-	-	-	-	[31]
Mazandaran	180	126 (70.0)	100	78	-	(78)	45	42 (93.33)	[15,23,32]
Semnan	50	40 (80)	-	-	-	-	-	-	[18]
Western Azerbaijan	206	71 (34.4)	-	-	-	-	-	-	[19]
Western Azerbaijan/Kurdistan/Kermanshah	83	74 (89.15)	-	-	22	21 (95.45)	10	10 (100)	[3]

=Study not conducted, No=Number of samples, Pos=Positive samples

## Discussion

The present study is the first systematic review of gastrointestinal parasitic infections of carnivores in Iran, providing accurate data for the prevalence of zoonotic parasites from 1997 to 2015. The overall prevalence rate of gastrointestinal parasitic infections among the studied carnivores in Iran was estimated to be 80% (97.32% jackals, 91.89% cats, 89.17% foxes, and 57.89% dogs).

The prevalence of intestinal parasitic infections in carnivorous has also been reported in other countries such as 90% in Sri Lanka [8], 71.33 % in Spain [35], and 17.6% in the Czech Republic [36]. These reported prevalence rates are variable and dependent on a number of factors including different detection methods, geographical climate (temperature and humidity), season, behaviors of the local animal populations, and the type of population of carnivores (stray, shelter, and household) [11,30].

Zoonotic pathogens can cause many different types of problem in human and animals ranging from mild-to-serious infection and even death. Zoonotic diseases are of particular concern for high-risk groups particularly children [37,38]. Carnivores, especially dogs and cats, act as the main reservoirs for many zoonotic diseases and play an important role in public health [39].

In Iran, domestic and stray dogs and cats carry the heaviest burden of zoonotic parasites [3]. In many parts of this country, foxes and jackals are also considered as potential sources of infection for humans. Although foxes and jackals generally live in forests and mountainous area, they have been reported proximal to human settlements [29]. As such these animals should be considered in monitoring programs as potential risks for zoonosis due to known interactions with regions of human habitat.

In this systematic review, the investigation of gastrointestinal parasites in carnivores revealed the significant prevalence of six critical zoonotic parasites in Iran including *T. canis, E. granulosus, D. caninum, M. lineatus, A. caninum*, and *M. hirudinaceus*. These parasites can cause serious clinical manifestations and diseases in human and should be considered as a major health problem.

### D. caninum

*D. caninum* is a common intestinal tapeworm of carnivorous that infects humans when they accidentally ingest infected fleas. Linnaeus reported the first known human case of dipylidiasis in 1758. There are more than 120 reports of human dipylidiasis in the world with the majority occurring in children due to either accidental ingestion of infected fleas or contact with saliva of pet animals [40,41]. In this study, *D. caninum* as a zoonotic helminths was the most predominant parasites species in cats (34.31%). Moreover, its prevalence was 32.21% in jackals, 16.89% in dogs, and 4.05% in foxes.

### M. lineatus

*M. lineatus* has a wide distribution in Asia, Europe, and Africa. Reports from Japan, China, and Korea indicate transmission of the parasite to humans can cause diarrhea [42]. In parts of Europe, the adult life stage of this tapeworm occurs with high incidence among foxes (up to 70%) but rarely in cats and dogs (Germany and Switzerland 2-4% and England 14%) [43]. In our study, foxes (51.98%) were the most infected animals with *M. lineatus* compared to jackals (39.28%), dogs (6.4%), and cats (4.69%).

### E. granulosus/multilocularis

*E. granulosus* is one of the major zoonotic parasitic infections in North Africa and the Middle East. Both *E. granulosus* and *E. multilocularis* have been reported from these areas [44,45]. There are three distinct cycles of *E. granulosus* in Iran:

1. Dogs and livestock (domestic cycle)
2. Dogs and camels (desert cycle)
3. Wild carnivores and wild ruminants (sylvatic cycle).

Hydatid cyst disease is more prevalent in Iran, and it is responsible for nearly 1% of all admission to surgical wards in Iran hospitals. The majority of cases of human and livestock hydatid cysts have been reported from the Khorasan Razavi Province [46]. Moreover, carnivores are considered as a definitive host for *E. multilocularis*, acquiring the infection from wild rodents, and can be a main zoonotic risk for alveolar cyst in humans [46,47].

Our review study suggests that the overall prevalence of *E. granulosus* in dogs, jackals, and foxes are 14.61%, 10.71%, and 1.8%, respectively. The global prevalence of echinococcosis in carnivores varies from 1% to 63.5% in East Africa, South Africa, South America, East Europe, and China [48,49].

A potential factor for the high prevalence of *E. granulosus* in Iran might be due to large populations of stray dogs and the lack of continuous comprehensive control programs, especially for farms and around towns [44,46].

### Toxocara spp.

*Toxocara* species (*T. cati, T. canis*, and *T. leonina*) are causative agents of visceral larva migrants in humans. The larva of these parasites can attack the eye cause ocular larva migrants and blindness. Children are the most frequently infected victims of these parasites owing to their close contact with contaminated soil [50]. According to the findings of the current study, the overall prevalence of *T. canis*/*cati* was 25.58%, 12.34%, 35.01%, and 22.32% for cats, dogs, foxes, and jackals, respectively. This considerable infection rate may increase the risk of zoonosis to humans and therefore may play an important role in human toxocariasis in Iran. The lowest and highest prevalence rates of this parasite in foxes were reported from Spain (4.4%) [51] and Denmark (81%) [52], respectively. In addition, the minimum and maximum infection rates of *T. canis* in dogs were reported from Czech Republic 6.2% and Slovak Republic 21.9%, respectively [36,53].

Whereas, the prevalence of *T. cati* was reported from other countries such as Estonia (48.2%) [54], Spain (55%) [55], and Turkey (62.5%) [56].

### M. hirudinaceus

*M. hirudinaceus* is an acanthocephalan known to infect humans and cause acanthocephaliasis. Although the definitive host for *M. hirudinaceus* is typically swine, carnivores and humans may act as accidental hosts [57]. Human cases of *M. hirudinaceus* have been reported from China [58] and Thailand [59]. In our study, 0.38% of dogs, 24.9% of foxes, and 5.3% of jackals were infected with the parasite. Although no human infections have been reported in Iran, this high infection rate could increase the possibility of acquiring infection to humans.

### A. caninum

*A. caninum* is one of the most pathogenic species in carnivores [29]. Larvae of *Ancylostoma* species cause cutaneous larva migrants or creeping eruption in human. Moreover, these nematodes are responsible for eosinophilic enteritis and unexplained abdominal pain with peripheral eosinophilia in humans [60]. In our study, the prevalence rate of *Ancylostoma* spp. was similar toxoascaris (8.77% and 8.69%, respectively). Due to the high prevalence of *A. caninum* in Jackals, they may be considered as the most important reservoir for cutaneous larva migrants in Iranian people.

## Conclusion

Based on the findings of the current study, the total prevalence of zoonotic intestinal parasites of carnivores in Iran is over 70%. Therefore, serious and continuous preventive measures should be taken into consideration owing to being a public concern in different provinces of Iran.

Control programs including prevention of environmental contamination with carnivore’s feces, reduction of the dog, cat, foxes, and jackal population, education program about the zoonotic potential of these parasites, and environmental and ecological modifications can reduce the risk of the transmission infection to human.

## Authors’ Contributions

SS, AD, MS, and SG conceptualized the concept of this review paper. MTR, MHK, and ABH statistical advisor and critically reviewed the manuscript. SG, SM, and AS prepared the manuscript. AS and SG assisted in collecting and compiling the resource material. All authors read and approved the final manuscript for publication.
